# Comparative analysis of body composition results of university students obtained using two bioimpedance analyzers

**DOI:** 10.2478/joeb-2025-0008

**Published:** 2025-05-06

**Authors:** Ma. Fidelina Peñaloza-Talavera, Clara H. Gonzalez-Correa, William Narvaez-Solarte, Izabella C. Gomes-Santana-Pereira, Jhony A. Diaz-Vallejo

**Affiliations:** Research Group in Nutrition, Metabolism, and Food Safety (NUTRIMESA), Universidad de Caldas, Colombia; Clinical nutrition Master's degree student, Montrer University, Mexico; Department of Basic Health Sciences, Universidad de Caldas; Department of Animal Health, Universidad de Caldas; Faculty of Medicine USP, Bauru campus, Brazil

**Keywords:** Bioimpedance, anthropometry, electrical impedance, body composition

## Abstract

Body composition is a key health indicator that distinguishes between fat mass and lean mass, factors associated with the risk of metabolic and cardiovascular diseases. Among measurement methods, bioelectrical impedance analysis (BIA) is widely used, with single- and multi-frequency devices showing differences in performance.

**Objective:**

To compare the accuracy of the OMRON^®^ HBF-514C (single-frequency) and BIODY XPERT ZM II^®^ (multi-frequency) bioimpedance analyzers in measuring body composition in university students.

**Materials and Methods:**

An observational, cross-sectional, and comparative study was con-ducted with 40 students (20 men, 20 women) from the University of Caldas, without a history of metabolic diseases. Body fat, muscle mass, basal metabolic rate (BMR), and BMI were measured using both devices. Data normality was assessed, and means were compared using the student’s t-test (p < 0.05).

**Results:**

In women, Biody showed significantly higher values for body fat, muscle mass, and basal metabolic rate compared to Omron (p < 0.05), with no differences in BMI. In men, only muscle mass and basal metabolic rate were significantly higher with Biody (p < 0.05), with no differences in body fat or BMI.

**Conclusions:**

Biody showed significantly higher values than Omron for muscle mass and basal metabolic rate. In women, the differences exceeded the acceptable 5% variability, suggesting that multifrequency devices may offer greater consistency. Although no gold standard was used, Omron could be a valid alternative in men.

## Introduction

Body composition refers to the proportions of the different components that make up the human body, focusing on the distribution of fat mass and fat-free mass, which includes muscles, bones, and other tissues. It is a fundamental aspect of health assessment because it provides more detailed information than traditional metrics such as body weight or body mass index (BMI), which do not differentiate between fat mass and lean mass [1].

Body composition plays a crucial role in health risk assessment. Excess fat, particularly visceral fat, is associated with cardiovascular and metabolic risks, as well as insulin resistance, increasing the likelihood of developing type 2 diabetes and cardiovascular disease [2,3,4]. Additionally, fat mass and fat-free mass have distinct associations with cancer risk, with excess fat being a risk factor for the development of diseases such as prostate, kidney, esophageal, and colon cancer [5]. Obesity, especially grade II obesity (on a classification scale of I–III), increases mortality risk, although this relationship decreases with advanced age [6,7]. An imbalance in body composition also contributes to impaired kidney function, sarcopenia, and osteoporosis, increasing the risk of fractures and functional loss with age [8].

There are various techniques for measuring body composition, each differing in precision and applicability. Common methods include anthropometry, with measurements of skinfolds, circumferences, and BMI, which are simple and non-invasive but less accurate [9]. Bioelectrical impedance analysis (BIA) is a popular method that uses the body's electrical resistance to calculate results; it is accessible and useful in population studies, although its accuracy varies compared to standard methods such as dual-energy X-ray absorptiometry (DXA), since its reliability decreases in individual assessments [10,11,12,13].

On the other hand, methods such as DXA, magnetic resonance imaging (MRI), computed tomography (CT), and air displacement plethysmography are more accurate and detailed, but their use is generally limited by cost, long wait times, the need for trained personnel, and potential radiation exposure [14,15,16]. The choice of methods depends on the purpose, population, and available resources, making it essential to understand the strengths and limitations of each technique.

Currently, new versions of equipment based on the principles of bioelectrical impedance have been developed, classified into three categories: single-frequency, multi-frequency, and electrical impedance spectroscopy. Devices that use more than two frequencies are an advanced tool in bioelectrical impedance analysis, as they employ a wide range of frequencies, typically up to 1,300 kHz, to provide more accurate measurements. These frequencies allow for a more precise distinction between intra- and extracellular water compartments, improving the validity of estimates of fat-free mass (FFM) and fat mass (FM). However, single-frequency devices, which operate with only one frequency, are more limited in this regard, as some authors argue that they cannot accurately discriminate between these compartments [17]. Despite their lower capacity, single-frequency devices offer a significant advantage as they are more accessible, less expensive, require less advanced technology to operate, and are more commonly used in biomedical research [18,19].

The differences between these devices may stem from variations in design, the technology used, the algorithms applied for calculations, adjustments in specific formulas, or the quality of the sensors, all of which affect measurement accuracy across devices. Therefore, it is essential to consider these variations when comparing results. Comparative analysis between these devices is crucial in research, as it helps identify their reliability and contributes to a better understanding of their usefulness in body composition assessment [20].

Thus, the objective of this study is to compare the agreement between the results of two bioimpedance analyzers: a low-cost, more accessible single-frequency device (Omron^®^) and a higher-cost multi-frequency device (Biody^®^), in measuring body composition in a sample of 40 students.

## Materials and methods

### Type of study

An observational, cross-sectional, quantitative, and comparative study was conducted with the objective of analyzing the agreement between two bioimpedance analyzers—one single-frequency and one multi-frequency—for measuring body composition in a selected sample of students.

### Population and sample

A convenience sample of 40 students from the University of Caldas (Colombia) was selected, with half being men and the other half women (20 women and 20 men). This was done to ensure balanced gender representation in the research. Each participant provided consent for the measurements to be taken.

### Inclusion criteria

Participants were selected based on the following criteria:

- Active students enrolled at the University of Caldas.
- Age between 18 and 30 years.
- No history of chronic diseases or medical conditions that could affect body composition measurements.
- No metabolic disorders that might interfere with the results obtained from the bioimpedance devices.

### Exclusion criteria

Individuals meeting any of the following conditions were excluded:

- Students who did not provide consent.
- Students with musculoskeletal disorders that could interfere with the measurement.
- Pregnant women.
- Individuals using medical electronic devices or metallic prostheses that could interfere with bioimpedance measurements.

### Bioimpedance analysis

Electrocardiogram electrodes of the 3M brand were used for the measurements. Regarding electrode placement, the specific instructions from the manufacturers for each device were followed. Measurements of body fat, muscle mass, basal metabolic rate, and body mass index were taken from all participants using two specific devices. Initially, the single-frequency device was used, followed by the multi-frequency device for all participants. Additionally, anthropometric measurements such as weight and height were recorded using a Seca digital scale and stadiometer, following the protocol by Lohman et al. [21]. Fasting state and physical activity were not considered, as the goal was to compare body composition results for everyone at the same time using two different devices, rather than to establish a diagnosis.

Devices:

- OMRON^®^ model HBF-514C (single-frequency), hereafter referred to as Device 1.
- BIODYXPERT ZM II^®^ (multi-frequency), hereafter referred to as Device 2.

Each measurement was performed according to the manufacturer's guidelines to ensure the validity of the results. Data were systematically recorded in an Excel database for subsequent comparative analysis.

### Statistical analysis

A normality test was conducted to determine whether the data followed a normal distribution. The means of the measurements obtained from both bioimpedance devices were compared using the paired *t*-test. A significance level of *p* < 0.05 was set to identify statistically significant differences between the results obtained from the two devices. All statistical analyses were performed using SPSS version 25.

### Informed consent

Informed consent has been obtained from all individuals included in this study.

### Ethical approval

The study was conducted in accordance with international biomedical research guidelines established in the Declaration of Helsinki [22]. It also complied with national regulations outlined in Resolution 8430 of 1993 [23].

## Results

Regarding anthropometric measurements, differences were observed in the characteristics between women and men (Table 1).

**Table 1.** General Measurements of the Population.

Variable	Women	Men
Age (years)	20.8 ± 2.13	22.20 ± 3.24
Body weight (kg)	56.52 ± 7.30	66.94 ± 7.61
Height (cm)	156.10 ± 4.55	172.55 ± 4.52
Waist circumference (cm)	74.42 ± 7.98	78.90 ± 6.75
Hip circumference (cm)	94.77 ± 4.48	93.80 ± 4.68

In the group of women, device 1 showed statistically lower means (p < 0.05) for body fat, muscle mass, and basal metabolic rate compared to measurements with device 2. As expected, there was no effect of the type of bioimpedance device (p < 0.05) on the Body Mass Index (Table 2).

**Table 2.** Comparison of the mean body condition of female university students measured with different bioimpedance devices (Mean±SD).

Bioimpedance Analyzer	Body Fat (%)	Muscle Mass (%)	Basal Metabolic Rate (kcal)	Body Mass Index (kg/m^2^)
OMRON	35.12±1.43	26.14±0.79	1248.65±18.83	23.25±0.71
BIODY	30.10±0.97	35.52±0.51	1359.50±12.23	23.19±0.70
P-value (p =)	**0.006**	**0.00**	**0.00**	0.95

In the group of male university students, the means of the variables evaluated with devices 1 vs. 2, using the Student t-test, showed that the percentages of muscle mass and basal metabolic rate were statistically higher (*p* < 0.05) when measured with device 2 compared to device 1. On the other hand, there was no significant difference (*p* < 0.05) between the means of body fat percentage and body mass index obtained with the two bioimpedance devices evaluated (Table 3).

**Table 3.** Comparison of the mean body composition of male university students measured with two different bioimpedance devices (Mean±SD).

Bioimpedance Analyzer	Body Fat (%)	Muscle Mass (%)	Basal Metabolic Rate (kcal)	Body Mass Index (kg/m^2^)
OMRON	17.55±1.23	41.69±0.74	1641.55±22.88	22.65±0.59
BIODY	16.87±1.03	45.24±0.60	1737.25±15.14	22.52±0.56
P-value (p =)	0.67	**0.001**	**0.00**	0.87

When comparing the means of body fat mass and hydrated fat mass, no statistically significant differences were identified between the two variables (p > 0.05). The recorded values were 23.48 ± 1.27 (body fat mass) and 23.05 ± 1.22 (hydrated fat mass), suggesting equivalence between the measurements. Additionally, an almost perfect correlation was observed between the two parameters, with a coefficient of r = 0.99, indicating a highly consistent linear relationship and near-total agreement in the results.

**Figure 1.**
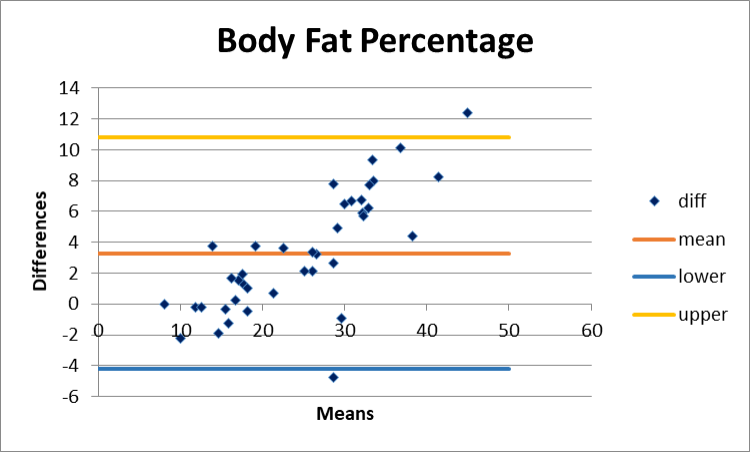
Bland & Altman graph for agreement between body fat percentage.

**Figure 2.**
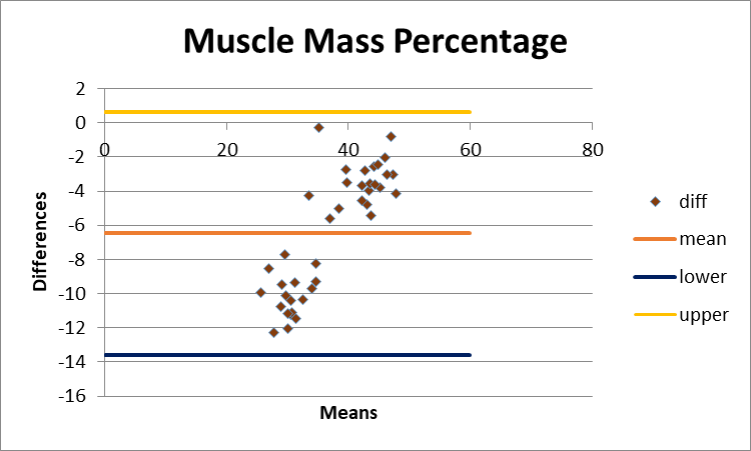
Bland & Altman graph for agreement between muscle mass percentage.

The results showed that the measurements of body fat, basal metabolic rate (BMR), and body mass index (BMI) taken by the two devices did not show agreement, as the differences were statistically significant according to the Bland-Altman test (p < 0.05), with a test power of 0.96 for body fat. In contrast, the muscle mass measurements showed agreement between the devices, with no significant differences (p > 0.05) and a test power of 0.94 (Figures 1–2).

## Discussion

The comparison between the two bioimpedance analyzers, Omron^®^ (single frequency) and Biody^®^ (multi-frequency), revealed significant differences in body composition measurements. The Bland-Altman analysis showed a lack of agreement between the devices for body fat percentage, basal metabolic rate (BMR), and body mass index (BMI), while muscle mass measurements demonstrated better concordance. These findings indicate that differences in technology, algorithmic estimations, and frequency range influence the precision and reliability of bioimpedance-based assessments.

The Bland-Altman plots demonstrated that Biody^®^ consistently reported higher values for muscle mass and BMR compared to Omron^®^, with mean biases exceeding clinically acceptable limits. These discrepancies suggest that single frequency bioimpedance devices may underestimate muscle mass and overestimate body fat due to their inability to differentiate between intra- and extracellular water compartments accurately, as supported by previous studies [24, 25].

The body fat percentage measurements showed significant variability between the two analyzers, particularly in women, where the difference exceeded the 5% threshold for acceptable variability in bioimpedance methods [26]. The observed bias may stem from differences in the estimation algorithms used by each manufacturer. The findings align with studies reporting that multi-frequency devices tend to provide lower body fat estimates and higher muscle mass values compared to single-frequency devices [27, 28].

Regarding BMI, the Bland-Altman analysis indicated no statistically significant differences between the devices, suggesting that both can provide similar results for this parameter. However, BMI does not differentiate between fat and lean mass, which may explain the stronger agreement despite discrepancies in other metrics. This finding aligns with previous reports stating that bioimpedance analyzers often yield consistent BMI values despite varying body composition estimates [29]. This finding could suggest that the discrepancies may be related to the calibration models of the devices. However, the measurements of muscle mass did show concordance between the devices, with no significant differences (p > 0.05) and a test power of 0.94. This reinforces the idea that a simple recalibration process between the devices could improve the consistency of the measurements, especially for parameters like body fat [30].

The muscle mass comparison demonstrated relatively better agreement between devices, particularly in men. However, Biody^®^ reported consistently higher muscle mass values, in line with the expectation that multi-frequency analyzers offer a more refined estimation of fat-free mass due to their enhanced ability to assess hydration status and intracellular composition [18]. Although the differences in muscle mass were statistically significant, they did not exceed the 5% variability threshold in men.

An essential factor to consider, especially for countries with limited financial resources, is the significant cost difference between devices. The Biody^®^ analyzer costs approximately 14 times more than the Omron^®^, creating a barrier to widespread clinical implementation. In contrast, more affordable devices like Omron^®^ could help democratize the use of bioelectrical impedance analysis (BIA) in clinical settings. This accessibility can play a critical role in enabling early diagnoses of conditions such as sarcopenia, especially in low-resource environments.

Equally important is the consistency in using the same bioimpedance device for longitudinal patient monitoring. Employing the same analyzer throughout follow-up assessments minimizes the impact of systematic errors inherent to each device, ensuring that changes in measurements more accurately reflect physiological variations rather than device-related discrepancies. This approach is particularly relevant in clinical settings where device precision varies, as it allows for reliable tracking of trends over time, even if the chosen device is not the most accurate. Ensuring consistency can thus improve clinical decision-making, particularly in the management of chronic conditions where body composition changes are gradual.

A limitation of the study is the lack of comparison with a gold standard, which could affect the accuracy of the results. Additionally, the small sample size of 40 participants limits the generalizability of the findings. Finally, the selection of healthy young individuals restricts the clinical applicability of the results, as it does not reflect the diversity of the general population, particularly individuals with comorbidities or various health conditions.

## Conclusions

In conclusion, while multi-frequency devices like Biody^®^ offer greater precision, single-frequency devices such as Omron^®^ provide a cost-effective alternative that could expand access to body composition assessments, especially in underserved communities. Consistency in using the same device for follow-up evaluations is critical for minimizing measurement variability and ensuring reliable patient monitoring. Future research should further explore the trade-offs between cost and precision and investigate strategies to optimize the clinical utility of affordable BIA devices.
